# Psychometric properties, pragmatic screening cut‐offs, and feasibility of two Clock Drawing Test formats in older psychiatric outpatients: Associations with MMSE and HDS‐R

**DOI:** 10.1002/pcn5.70351

**Published:** 2026-05-25

**Authors:** Takayo Hatasaki, Norio Sugawara, Yasushi Kawamata, Chie Hasegawa, Norio Yasui‐Furukori

**Affiliations:** ^1^ Department of Psychiatry Dokkyo Medical University Shimotsuga Japan

**Keywords:** Clock Drawing Test, cognitive screening, feasibility, HDS‐R, MMSE, psychometrics

## Abstract

**Aim:**

To compare the psychometric properties, pragmatic screening cut‐offs, and feasibility of two Clock Drawing Test (CDT) formats in older psychiatric outpatients.

**Methods:**

We retrospectively analyzed 447 outpatients aged ≥65 years who completed the Mini‐Mental State Examination (MMSE), the Hasegawa Dementia Scale‐Revised (HDS‐R), and two CDT formats scored using the Freedman method: a free‐drawn clock (CDT1) and an examiner‐provided clock face (CDT2). Pearson correlations, forced‐entry multiple regression models, exploratory factor analyses, and receiver operating characteristic (ROC) analyses were used. Possible cognitive impairment was operationally defined as MMSE ≤ 23 and/or HDS‐R ≤ 20. Administration time was summarized in a convenience subsample (*n* = 20).

**Results:**

Mean (SD) age was 80.20 (5.15) years, and mean education was 11.74 (2.36) years. CDT totals correlated positively with MMSE and HDS‐R totals (*r* = 0.47–0.58, all *p* < 0.01). Education, functional disability, CDT1, and CDT2 predicted MMSE total, while age additionally predicted HDS‐R total. Both CDT formats showed interpretable two‐factor structures with good internal consistency (Cronbach's *α* 0.82–0.90). ROC analyses identified the same pragmatic cut‐off (10/11) for both formats (CDT1 area under the curve [AUC] 0.72, 95% CI 0.67–0.77; CDT2 AUC 0.71, 95% CI 0.66–0.76). Mean CDT administration time was 2.13 min.

**Conclusion:**

Both CDT formats showed clinically useful psychometric properties and similar pragmatic cut‐offs in older psychiatric outpatients. CDT1 favored sensitivity, and CDT2 favored specificity at the same threshold. The CDT was highly feasible in routine practice, requiring approximately 2 min to administer.

## INTRODUCTION

Population ageing has made dementia a major public health and socioeconomic challenge. The GBD 2019 Dementia Forecasting Collaborators estimated 57.4 million people living with dementia worldwide in 2019 and projected the number to exceed 150 million by 2050.^1^ Japan, one of the most rapidly ageing societies, has documented secular increases in dementia prevalence in population‐based studies such as the Hisayama Study.^2^, ^3^ In parallel with advances in diagnosis and treatment, prevention and risk reduction across the life course have become central public health priorities, as highlighted by the Lancet Commission and the development of WHO guidance.^4^, ^5^, ^6^

Mild cognitive impairment (MCI) refers to cognitive decline beyond normal ageing, with relative preservation of independence in basic activities, and is a high‐risk state for progression to dementia.^7^, ^8^, ^9^ In geriatric psychiatry, early recognition of cognitive impairment is particularly important because affective symptoms, psychosis, sleep disturbance, and medication effects can overlap with neurocognitive disorders. Timely detection facilitates targeted diagnostic work‐up, risk‐factor modification, and care planning, including linkage to community‐based services.

In routine outpatient practice, brief global cognitive screeners remain the cornerstone of initial assessment. The Mini‐Mental State Examination (MMSE), introduced by Folstein et al.,^10^ and the Hasegawa Dementia Scale‐Revised (HDS‐R), widely used in Japan,^11^ provide efficient snapshots of orientation, memory, and language. However, both instruments show well‐described influences of age and education and may be less sensitive to subtle executive and visuospatial dysfunction, especially in highly educated individuals or in non‐amnestic presentations.^12^, ^13^

Because dementia is characterized by cognitive decline that interferes with everyday function and often co‐occurs with behavioral and psychological symptoms of dementia (BPSD), comprehensive evaluation benefits from brief parallel measures of functional and neuropsychiatric status. The Dementia Assessment Sheet for Community‐based Integrated Care System (DASC‐21) was developed in Japan to assess cognitive and daily functional impairment, with evidence for reliability and discriminant validity against clinician‐rated dementia staging^14^; a short‐form (DASC‐8) has also been validated for efficient screening.^15^ Neuropsychiatric symptoms can be quantified using instruments such as the Neuropsychiatric Inventory (NPI) and shorter questionnaires designed for routine care settings.^16^, ^17^

Among brief neuropsychological tasks, the Clock Drawing Test (CDT) is widely used because it is time‐efficient, well accepted by older adults, and captures multiple cognitive operations—including semantic knowledge, visuoconstruction, planning, and executive monitoring—within a single drawing.^18^, ^19^, ^20^ Nevertheless, systematic reviews emphasize that the CDT is not standardized: administration procedures (e.g., free‐drawn vs. examiner‐provided clock face; command vs. copy) and scoring systems vary substantially, which affects psychometric properties and limits cross‐study comparability.^20^, ^21^

One structured approach to CDT scoring is the Freedman method, which provides component‐based quantitative scoring intended to support neuropsychological interpretation.^22^ Despite decades of CDT research, there remains limited evidence on how different administration formats behave psychometrically in real‐world geriatric psychiatry outpatient settings, and few studies have examined item‐level factor structures alongside both MMSE and HDS‐R in Japanese clinical practice. Accordingly, we conducted a retrospective observational study in older psychiatric outpatients to (1) examine associations between two CDT formats and global cognitive screeners (MMSE and HDS‐R), (2) evaluate demographic and functional correlates of MMSE and HDS‐R scores, (3) investigate the factor structure and internal consistency of each CDT format scored using the Freedman method, and (4) explore practical screening cut‐offs via receiver operating characteristic (ROC) analysis. Given the lack of standardized diagnostic adjudication in routine outpatient screening, we explicitly conceptualized ROC analyses as pragmatic operational alignment with commonly used screening thresholds rather than definitive diagnostic accuracy estimates. The purpose of the ROC analysis was not to evaluate CDT as a replacement for MMSE or HDS‐R, but to examine how CDT performance aligns with and complements these widely used screening instruments in routine outpatient practice.

## METHODS

### Study design and setting

This retrospective observational study was conducted at a psychiatric outpatient clinic in Japan, where brief cognitive screening is routinely performed for older adults presenting with cognitive complaints, caregiver concerns, or suspected neurocognitive disorders. The study evaluated two CDT administration formats scored using the Freedman method and their psychometric properties in relation to global cognitive screening measures.

### Participants

Participants were 447 outpatients aged 65 years or older (183 men, 264 women) who visited the clinic between April 2022 and May 2025 and completed the MMSE, HDS‐R, and both CDT formats during routine assessment. We included consecutive patients with complete data for these measures; patients with missing test data were excluded from analysis. Because this was a real‐world psychiatric outpatient sample, primary psychiatric diagnoses and medication exposures were heterogeneous; these factors were not modeled in the primary analyses and are addressed as limitations.

### Measures

Demographic and clinical variables included sex, age (years), years of education, and long‐term care insurance certification level (a proxy of care needs in the Japanese long‐term care system).

Global cognitive function was assessed using the MMSE and the HDS‐R. The MMSE is a 30‐point global cognitive screening instrument that assesses orientation, registration, attention and calculation, recall, language, and visuoconstruction. Lower scores indicate poorer cognitive performance; scores of 23 or lower are commonly used to indicate possible dementia or clinically significant cognitive impairment. The reliability and validity of the MMSE have been widely reported in older adults.^10^, ^12^, ^13^

The HDS‐R is a 30‐point cognitive screening scale widely used in Japan. It assesses orientation, immediate and delayed recall, attention/working memory, and verbal fluency. Lower scores indicate poorer cognitive performance; a score of 20 or lower is commonly used as a threshold for suspected dementia. The HDS‐R has demonstrated clinical utility, reliability, and validity in Japanese older adults.^11^

Functional status (“functional disability”) was assessed using the DASC‐21, with higher scores indicating greater impairment in cognition and daily functioning. The DASC‐21 was developed and validated in Japan as a brief assessment of cognitive and daily functional impairment for community‐based and clinical care settings.^14^, ^15^

Behavioral and psychological symptoms were assessed in routine practice using brief questionnaires, including DBP13/BPSD13Q, when clinically indicated. These measures were used to support routine clinical evaluation but were not included in the primary analyses of the present study.

A hybrid cognitive screening tool was used in routine clinical practice to reduce administration time by allowing the MMSE and HDS‐R to be administered concurrently. The MMSE and HDS‐R are both established 30‐point cognitive screening instruments that are widely used in Japan and have been included together as standard comparative measures in studies of cognitive screening and dementia assessment.^10^, ^11^, ^12^, ^13^, ^23^, ^24^, ^25^ The hybrid tool was designed to avoid repeatedly administering overlapping items shared by the MMSE and HDS‐R, thereby reducing redundancy while preserving the core screening components of both instruments. In the present study, the hybrid tool was used only as a feasibility comparator for administration time and was not analyzed as an independent diagnostic scale.

Two CDT formats were administered and scored using the Freedman method. CDT1 (free‐drawn clock) required drawing a clock with numerals 1–12 and setting the hands to “15 min to 7” (6:45). CDT2 (examiner clock) used a pre‐drawn circle with numerals and required drawing hands to show 11:10. CDT1 was scored on a 0–15 scale and CDT2 on a 0–11 scale, with each criterion scored dichotomously (0/1). Because CDT administration and scoring methods vary substantially across studies, there is no single universally accepted cut‐off. Therefore, this study examined format‐specific pragmatic cut‐offs aligned with established MMSE and HDS‐R screening thresholds.^18^, ^19^, ^20^, ^21^, ^22^

### Statistical analysis

Pearson correlation coefficients were computed among demographic variables, functional disability, and cognitive test scores. Because correlation matrices and subdomain‐level analyses involve multiple statistical tests, these analyses were interpreted as exploratory, and nominal (two‐tailed) *p*‐values are reported. Forced‐entry multiple regression models examined predictors of MMSE and HDS‐R total scores (sex, age, education, long‐term care certification level, functional disability, CDT1 total, and CDT2 total). Model diagnostics included assessment of multicollinearity (variance inflation factors) and inspection of residual plots.

DBP13/BPSD13Q scores were not included in the primary regression models because these data were collected during routine clinical practice when clinically indicated and were not systematically available for all participants. Including these measures would have reduced the analytic sample and increased the risk of overfitting. Moreover, the primary aim of this study was to evaluate the relationship between CDT performance and cognitive screening measures rather than behavioral and psychological symptoms.

Exploratory factor analyses were conducted separately for each CDT format using item‐level (0/1) scores to examine factor structure; factors were retained based on eigenvalues, scree plots, and interpretability. Because Freedman criteria are dichotomous, factor analytic results should be interpreted descriptively; replication using categorical‐data factor analytic methods (e.g., tetrachoric correlations) is warranted. Internal consistency of each factor was evaluated using Cronbach's alpha. Correlations between CDT factors and MMSE/HDS‐R subdomains were examined.

ROC analyses were conducted to examine how CDT performance aligns with and complements established MMSE and HDS‐R screening thresholds in routine outpatient practice. The ROC analysis was not intended to demonstrate that CDT could replace MMSE or HDS‐R, nor was it intended to establish diagnostic accuracy against clinician‐adjudicated dementia diagnoses. Possible cognitive impairment was operationally defined a priori as meeting either MMSE ≤ 23 or HDS‐R ≤ 20. Cut‐offs were selected to optimize the balance between sensitivity and specificity.

Administration time was evaluated as an exploratory feasibility outcome in a convenience subsample of 20 participants. This subsample consisted of patients for whom detailed timing data were recorded by trained staff during routine clinical assessments. The subsample was based on the availability of administration‐time records rather than formal matching or selection by age, diagnosis, cognitive status, or test performance.

### Ethical considerations

The study protocol was reviewed and approved by the Ethics Committee of Dokkyo Medical University Hospital (approval number: R‐34‐6J). Given the retrospective observational design and the use of anonymized clinical data obtained during routine clinical practice, the requirement for written informed consent was waived by the ethics committee in accordance with institutional guidelines and national regulations.

## RESULTS

Results are summarized below with supporting tables. Main findings are presented in Tables 1, 2, 3, 4, and detailed exploratory analyses are provided in the Supplementary Material (Tables S1–S4).

**Table 1:** Participant characteristics, cognitive test scores, and administration time.

Variable	Mean (SD)/*n* (%)
Age, years	80.20 (5.15)
Sex (male)	183 (40.9%)
Years of education	11.74 (2.36)
Long‐term care certification level	1.04 (1.83)
Functional disability score	50.06 (19.62)
MMSE total score (0–30)	21.38 (5.30)
HDS‐R total score (0–30)	19.13 (6.33)
CDT1 total score (0–15)	10.03 (4.36)
CDT2 total score (0–11)	8.71 (2.96)
CDT administration time, min (*n* = 20)	2.13 (1.43)
Hybrid screening time, min (*n* = 20)	10.2 (2.67)

*Note*: Administration time was available in a convenience subsample (*n* = 20). Values are mean (SD) unless otherwise indicated.

Abbreviations: CDT, Clock Drawing Test; HDS‐R, Hasegawa Dementia Scale‐Revised; MMSE, Mini‐Mental State Examination.

### Participant characteristics and test performance

A total of 447 psychiatric outpatients aged ≥65 years (183 men and 264 women) were included in the analyses. The mean age was 80.20 years (SD 5.15), and the mean years of education was 11.74 (SD 2.36). The mean long‐term care certification level was 1.04 (SD 1.83), and the mean functional disability score was 50.06 (SD 19.62).

Global cognitive screening indicated mild‐to‐moderate impairment on average (MMSE mean 21.38, SD 5.30; HDS‐R mean 19.13, SD 6.33). CDT performance showed a wide distribution for both formats (CDT1 mean 10.03, SD 4.36; CDT2 mean 8.71, SD 2.96). Functional disability suggested substantial heterogeneity in everyday functioning within this outpatient cohort (Table 1).

### Feasibility: Administration time

In the convenience subsample with available timing data (*n* = 20), CDT administration required a mean of 2.13 min (SD 1.43; median 2.0; range 1.0–7.0). In comparison, the hybrid MMSE/HDS‐R screening tool required a mean of 10.2 min (SD 2.67; median 10.0; range 6.0–16.0). These findings support the feasibility of CDT as a very brief screening adjunct in routine outpatient settings. Because the timing analysis was based on a convenience subsample, these results should be interpreted as exploratory.

### Correlations among demographic, functional, and cognitive measures

Bivariate correlations among demographic variables, functional disability, and cognitive measures are shown in Table 2. Age was negatively correlated with HDS‐R (*r* = −0.16, *p* < 0.01), MMSE (*r* = −0.12, *p* < 0.05), CDT1 (*r* = −0.19, *p* < 0.01), and CDT2 (*r* = −0.17, *p* < 0.01). Education showed positive correlations with MMSE (*r* = 0.30, *p* < 0.01), HDS‐R (*r* = 0.21, *p* < 0.01), CDT1 (*r* = 0.33, *p* < 0.01), and CDT2 (*r* = 0.28, *p* < 0.01).

**Table 2:** Pearson correlations among demographic variables, functional disability, and cognitive measures.

Variable	MMSE	HDS‐R	CDT1	CDT2
Age	−0.12*	−0.16**	−0.19**	−0.17**
Years of education	0.30**	0.21**	0.33**	0.28**
Functional disability	−0.51**	−0.50**	−0.43**	−0.34**
MMSE	–	0.74**	0.58**	0.53**
HDS‐R		–	0.54**	0.47**
CDT1			–	0.59**

*Note*: Pearson correlation coefficients are shown. Analyses were exploratory.

Abbreviations: CDT, Clock Drawing Test; HDS‐R, Hasegawa Dementia Scale‐Revised; MMSE, Mini‐Mental State Examination.

* *p* < 0.05

** *p* < 0.01.

**Table 3:** Multiple regression analyses predicting Mini‐Mental State Examination (MMSE) and Hasegawa Dementia Scale‐Revised (HDS‐R) total scores.

Predictor	MMSE (*β*)	HDS‐R (*β*)
Sex	−0.04	0.04
Age	−0.02	−0.09*
Years of education	0.19***	0.10*
Long‐term care certification	−0.01	−0.04
Functional disability	−0.28***	−0.29***
CDT1 total score	0.29***	0.29***
CDT2 total score	0.23***	0.17***
*R* ^2^	0.50***	0.44***
Adjusted *R* ^2^	0.49***	0.43***

*Note*: Standardized regression coefficients (*β*) from forced‐entry models.

Abbreviation: CDT, Clock Drawing Test.

* *p* < 0.05

*** *p* < 0.001.

**Table 4:** Receiver operating characteristic (ROC) characteristics and screening cut‐off values of Clock Drawing Test (CDT)1 and CDT2.

Measure	AUC (95% CI)	Cut‐off	Sensitivity	Specificity
CDT1 (free‐drawn clock, 0–15)	0.72 (0.67–0.77)	10/11	0.82	0.55
CDT2 (examiner‐provided clock, 0–11)	0.71 (0.66–0.76)	10/11	0.57	0.75

*Note*: Cut‐off values were selected to optimize the balance between sensitivity and specificity. Reference standard: Mini‐Mental State Examination (MMSE) ≤ 23 and/or Hasegawa Dementia Scale‐Revised (HDS‐R) ≤ 20.

Abbreviation: AUC, area under the curve.

Functional disability demonstrated moderate negative correlations with HDS‐R (*r* = −0.50, *p* < 0.01), MMSE (*r* = −0.51, *p* < 0.01), CDT1 (*r* = −0.43, *p* < 0.01), and CDT2 (*r* = −0.34, *p* < 0.01). MMSE and HDS‐R totals were strongly correlated (*r* = 0.74, *p* < 0.01). Both CDT formats correlated positively with MMSE (CDT1 *r* = 0.58; CDT2 *r* = 0.53; both *p* < 0.01) and HDS‐R (CDT1 *r* = 0.54; CDT2 *r* = 0.47; both *p* < 0.01). The two CDT totals were moderately correlated with each other (*r* = 0.59, *p* < 0.01).

### Predictors of global cognitive screening scores

Forced‐entry multiple regression models were used to identify independent predictors of MMSE and HDS‐R total scores (Table 3). For MMSE, education (*β* = 0.19, *p* < 0.001), functional disability (*β* = −0.28, *p* < 0.001), CDT1 total score (*β* = 0.29, *p* < 0.001), and CDT2 total score (*β* = 0.23, *p* < 0.001) were significant predictors, whereas sex, age, and long‐term care certification level were not independently associated with MMSE performance.

For HDS‐R, age (*β* = −0.09, *p* < 0.05), education (*β* = 0.10, *p* < 0.05), functional disability (*β* = −0.29, *p* < 0.001), CDT1 total score (*β* = 0.29, *p* < 0.001), and CDT2 total score (*β* = 0.17, *p* < 0.001) were significant predictors. The regression models explained a substantial proportion of variance in global cognitive performance (MMSE adjusted *R* ^2^ = 0.49; HDS‐R adjusted *R* ^2^ = 0.43).

### ROC analysis for practical screening cut‐offs

**Figure 1:**
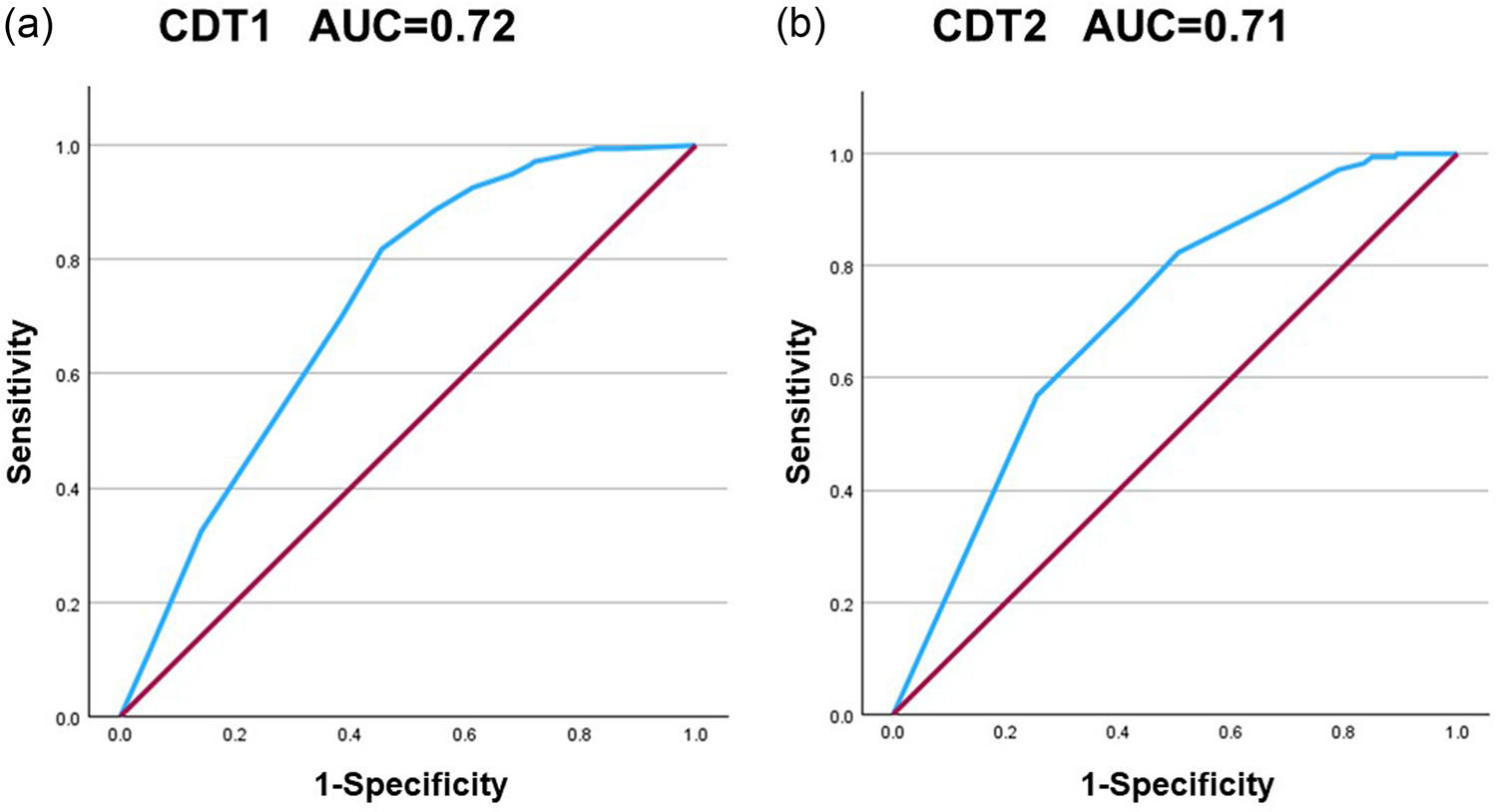
Receiver operating characteristic (ROC) curves for two Clock Drawing Test (CDT) formats scored by the Freedman method: (a) CDT1 (free‐drawn clock) and (b) CDT2 (examiner‐provided clock face). The reference standard for possible cognitive impairment was Mini‐Mental State Examination (MMSE) ≤ 23 and/or Hasegawa Dementia Scale‐Revised (HDS‐R) ≤ 20. AUC, area under the curve.

ROC analyses were conducted to explore practical CDT thresholds for classifying possible cognitive impairment, operationally defined as meeting either MMSE ≤ 23 or HDS‐R ≤ 20. These analyses were intended to evaluate the complementary alignment of CDT with established screening instruments rather than to test whether CDT could replace MMSE or HDS‐R. For CDT1, discrimination was modest (AUC 0.72, 95% CI 0.67–0.77), and an optimal cut‐off of 10/11 yielded high sensitivity (0.82) with moderate specificity (0.55). For CDT2, discrimination was similarly modest (AUC 0.71, 95% CI 0.66–0.76), and the same cut‐off of 10/11 yielded lower sensitivity (0.57) but higher specificity (0.75). Thus, while overall discrimination was comparable across formats, CDT1 favored sensitivity, whereas CDT2 favored specificity at the same threshold (Table 4). The corresponding ROC curves are shown in Figure 1.

### Exploratory factor structure and subdomain associations (Supplementary Material)

Exploratory factor analyses supported two‐factor solutions for both CDT formats with good internal consistency (Cronbach's *α* = 0.82–0.90). For CDT1, Factor 1 primarily reflected hand‐related perceptual/executive organization, including the presence and connection of clock hands and center‐point placement. Factor 2 primarily reflected numeral‐related visuospatial placement, including numeral format, order, and spatial arrangement within the clock face. For CDT2, Factor 1 reflected structural integrity and spatial anchoring, including center‐point stability, hand connection, and absence of extraneous marks. Factor 2 reflected the accuracy of time representation, including angular accuracy and correct indication of the target time. Full item‐level factor loadings are provided in Tables S1 and S2. Associations between CDT factor scores and MMSE/HDS‐R subdomains, and exploratory regression models identifying subdomain predictors of CDT factor scores, are provided in Tables S3 and S4.

## DISCUSSION

In this sample of older psychiatric outpatients, both CDT formats showed moderate associations with global cognitive screening measures (MMSE and HDS‐R), and each format demonstrated a stable two‐factor structure when scored using the Freedman method. Our findings are consistent with early clinical studies reporting that CDT performance tracks dementia severity and correlates with global cognition.^26^, ^27^, ^28^, ^29^

Importantly, the CDT is a family of tasks rather than a single standardized instrument. Seminal work proposed structured scoring criteria to capture errors in clock face integrity, numeral placement, and hand setting,^30^, ^31^, ^32^ and the CLOX emphasized executive‐control demands by separating command and copy conditions.^33^ Against this background, our observation that CDT1 (free‐drawn clock) and CDT2 (examiner‐provided clock face) share a similar screening cut‐off yet differ in factor composition suggests that the cognitive demands of “drawing a clock” depend strongly on how the task is administered and what is being scored.

For CDT1, the two‐factor solution broadly separated a perceptual/executive organization factor from a visuospatial placement factor. Conceptually, clock drawing requires integration of semantic knowledge, visuospatial construction, and planning/executive monitoring; component‐based scoring can help distinguish errors arising from executive dyscontrol versus constructional failure.^33^ Longitudinal community studies also suggest that declining clock performance can precede or predict subsequent cognitive decline, supporting the clinical relevance of component‐level analyses rather than reliance on a total score alone.^34^, ^35^

For CDT2, providing a pre‐drawn circle and numerals likely reduces demands on initial visuoconstruction and spatial planning, thereby shifting relative weight toward hand placement, time representation, and error monitoring. This may explain why CDT2 showed a somewhat different pattern of correlations with MMSE and HDS‐R subdomains in our analyses, despite comparable overall screening performance.

Our regression models indicated that education and functional disability were independently associated with MMSE and HDS‐R totals, and these factors also related to CDT performance. Education‐related differences are well documented for brief global cognitive screeners,^12^, ^13^ and prior reviews emphasize that CDT performance is sensitive to demographic and contextual influences as well as to the chosen scoring system.^20^, ^21^ These findings argue against interpreting CDT scores in isolation; instead, scores should be integrated with educational history and functional context in outpatient decision‐making.

Neurobiological studies provide convergent validity for the CDT as a multidomain task. In Alzheimer's disease, neuroimaging work has linked poorer CDT performance to dysfunction in distributed networks involving parietal and frontal regions implicated in visuospatial processing and executive control.^36^ This is consistent with our finding that CDT factors reflecting organization/planning and spatial placement were differentially associated with cognitive subdomains.

A key finding of this study is that two CDT administration formats yielded an identical pragmatic cut‐off (10/11) when aligned with established screening thresholds, despite differences in operating characteristics. At the same threshold, CDT1 emphasized sensitivity (0.82) whereas CDT2 emphasized specificity (0.75), suggesting that the two formats may serve complementary roles depending on clinical priorities (e.g., triage vs. minimizing false positives in time‐limited settings). These findings support the interpretation of CDT as a complementary screening adjunct rather than a replacement for MMSE or HDS‐R.

Importantly, ROC analyses in the present study were not intended to establish diagnostic accuracy against clinician‐adjudicated dementia diagnoses. Rather, they provide an operational alignment of CDT scores with commonly used screening instruments (MMSE and HDS‐R) in routine psychiatric outpatient practice. This distinction is critical because the use of screening thresholds as a reference standard introduces conceptual circularity and necessitates cautious interpretation of AUC values. External validation against standardized diagnostic adjudication and longitudinal follow‐up is warranted. As shown in Figure 1, both CDT formats exhibited modest discrimination with a shared pragmatic cut‐off, but with complementary sensitivity–specificity profiles, supporting format selection according to clinical priorities.

Beyond psychometric performance, CDT demonstrated high feasibility in routine outpatient practice. In a convenience subsample, mean administration time was 2.13 min (SD 1.43; median 2.0; range 1.0–7.0), whereas the hybrid MMSE/HDS‐R screening tool required 10.2 min on average (SD 2.67; median 10.0; range 6.0–16.0). Although these feasibility findings are exploratory, they strengthen the rationale for implementing CDT—particularly the examiner‐provided format—as a practical adjunct in busy geriatric psychiatry settings where time constraints often limit comprehensive cognitive assessment. From a clinical perspective, CDT2 may offer a pragmatic option because it preserves the hand‐setting component that stresses executive monitoring while reducing the motor and visuoconstruction demands of drawing the clock face. When used within a stepped screening pathway—combined with brief global cognition tests and targeted functional/BPSD measures—it may facilitate earlier identification of patients who warrant comprehensive neuropsychological evaluation and multidisciplinary care planning.

Several limitations should be considered. First, the retrospective single‐center design and inclusion of psychiatric outpatients limit generalizability; future studies should examine multisite samples and report diagnostic strata (e.g., depressive disorders, psychotic disorders, and major and mild neurocognitive disorders). Second, our ROC analyses used an operational reference standard based on MMSE/HDS‐R screening thresholds rather than clinician‐adjudicated diagnoses, which introduces conceptual circularity; therefore, ROC results should be interpreted cautiously and should be replicated using standardized diagnostic work‐up and longitudinal outcomes. Third, Freedman scoring involves rater judgment; inter‐rater reliability was not formally assessed in this dataset and should be reported in future implementation studies. Fourth, CDT item scores are dichotomous, and factor analyses based on Pearson correlations may yield biased parameter estimates; while the observed two‐factor solutions were interpretable, replication using categorical‐data factor analytic methods (e.g., tetrachoric correlations and confirmatory factor analysis) is needed. In addition, under oblique rotations, pattern coefficients may exceed 1.0 and should not be interpreted as simple correlations; we report these coefficients as descriptive indices of factor structure. Fifth, potential confounding from mood symptoms, psychotropic medications (including anticholinergic burden and sedatives), sensory impairment, and motor disorders was not modeled and may have influenced CDT performance. Prior Japanese work using Freedman scoring in schizophrenia has shown that both disease and aging affect CDT performance, reinforcing the need to consider psychiatric and demographic confounders when interpreting CDT scores.^37^ Sixth, administration time was assessed only in a convenience subsample rather than a formal time‐motion study and may have been influenced by age, cognitive severity, psychiatric symptoms, motivation, and clinical workflow; feasibility estimates should therefore be interpreted as preliminary and confirmed in prospectively timed samples. Finally, several analyses (correlations and subdomain models) were exploratory and involved multiple statistical tests; replication in independent datasets is needed to confirm the robustness of secondary findings.

In conclusion, two CDT administration formats scored with the Freedman method showed comparable screening utility and revealed interpretable two‐factor structures in older psychiatric outpatients. These results support the CDT as a practical adjunct to MMSE/HDS‐R in geriatric psychiatry, while emphasizing the need for setting‐specific validation and integration with functional and neuropsychiatric assessments.

## CONCLUSIONS

Two CDT formats scored with the Freedman method showed meaningful associations with MMSE and HDS‐R and interpretable two‐factor structures in older psychiatric outpatients. Using pragmatic screening thresholds as a reference, an operational cut‐off of 10/11 may be useful for intake screening. Given its higher specificity at the same cut‐off, the examiner‐clock format (CDT2) may be particularly suitable for routine screening when false positives are a concern. Further validation against clinician‐adjudicated diagnoses and longitudinal outcomes is required.

## AUTHOR CONTRIBUTIONS

Takayo Hatasaki contributed to data collection and data management. Norio Sugawara contributed to study design and interpretation of the data. Yasushi Kawamata contributed to clinical supervision and interpretation of the results. Chie Hasegawa contributed to data interpretation and critical revision of the manuscript. Norio Yasui‐Furukori conceived and designed the study, performed the statistical analyses, interpreted the data, and drafted the manuscript. All authors read and approved the final manuscript.

## CONFLICT OF INTEREST STATEMENT

The authors declare no conflicts of interest.

## ETHICS APPROVAL STATEMENT

This study was reviewed and approved by the Ethics Committee of Dokkyo Medical University Hospital (approval number: R‐34‐6J). Given the retrospective observational design and the use of anonymized clinical data obtained during routine clinical practice, the requirement for written informed consent was waived by the ethics committee in accordance with institutional guidelines and national regulations.

## PATIENT CONSENT STATEMENT

Patient consent was waived by the Ethics Committee because this study used retrospective anonymized clinical data.

## CLINICAL TRIAL REGISTRATION

N/A.

## Supporting information

Supporting Information.

## Data Availability

The data that support the findings of this study are available on request from the corresponding author. The data are not publicly available due to privacy or ethical restrictions.
