# Supplementary material for: Psychometric properties, pragmatic screening cut‐offs, and feasibility of two Clock Drawing Test formats in older psychiatric outpatients: Associations with MMSE and HDS‐R
Source: PCN Rep. 2026 May 25;5(2):e70351. doi: 10.1002/pcn5.70351 (PMC13240572; doi:10.1002/pcn5.70351)
Supplement: Supplementary file 1 — Supporting Information. [file PCN5-5-e70351-s001.docx]

**S1. Factor loadings for CDT1 (free-drawn clock) using Freedman scoring**

| **Item (Freedman)** | **Description** | **Factor 1** | **Factor 2** |
| --- | --- | --- | --- |
| 9 | Two hands present | 0.97 | −0.01 |
| 14 | Hands intersect/connected | 0.90 | −0.01 |
| 15 | Center point present | 0.89 | 0.06 |
| 13 | No extra marks | 0.52 | 0.14 |
| 10 | Hour hand correct | 0.50 | 0.17 |
| 12 | Minute hand longer | 0.47 | 0.01 |
| 11 | Minute hand correct | 0.37 | 0.05 |
| 4 | Arabic numerals used | −0.08 | 1.00 |
| 6 | No paper rotation | −0.06 | 0.97 |
| 5 | Numerals in correct order | 0.21 | 0.47 |
| 8 | Numerals inside circle | 0.14 | 0.42 |
| 3 | Numerals 1–12 present | 0.13 | 0.40 |
| 2 | Clock face shape adequate | 0.28 | 0.36 |
| 7 | Numeral positions correct | 0.25 | 0.29 |
| 1 | Circle size appropriate | 0.21 | 0.29 |

**Note:** Two-factor solution with oblique rotation.
Cronbach’s α = 0.87 for both factors.

**S2. Factor loadings for CDT2 (examiner-provided clock) using Freedman scoring**

| **Item** | **Description** | **Factor 1** | **Factor 2** |
| --- | --- | --- | --- |
| 9 | Center point present | 0.95 | −0.09 |
| 1 | Two hands present | 0.85 | 0.03 |
| 10 | Center not shifted left/right | 0.80 | −0.01 |
| 8 | Hands intersect/connected | 0.68 | −0.03 |
| 11 | Center not shifted up/down | 0.65 | 0.05 |
| 7 | No extra marks | 0.60 | 0.02 |
| 2 | Hour hand correct | 0.58 | 0.21 |
| 6 | Minute hand within 10° | −0.16 | 1.07 |
| 5 | Hour hand within 10° | 0.05 | 0.77 |
| 3 | Minute hand correct | 0.27 | 0.59 |
| 4 | Minute hand longer | 0.05 | 0.52 |

**Note:** Two-factor solution with oblique rotation.
Cronbach’s α = 0.90 (Factor 1), 0.82 (Factor 2).

**S3. Correlations between CDT factor scores and MMSE/HDS-R subdomains**

| **Cognitive subdomain** | **CDT1 Factor 1** | **CDT1 Factor 2** | **CDT2 Factor 1** | **CDT2 Factor 2** |
| --- | --- | --- | --- | --- |
| MMSE orientation | 0.42** | 0.39** | 0.38** | 0.33** |
| MMSE memory | 0.47** | 0.41** | 0.42** | 0.37** |
| MMSE attention/calculation | 0.35** | 0.31** | 0.29** | 0.26** |
| HDS-R recall | 0.45** | 0.40** | 0.41** | 0.36** |
| HDS-R verbal fluency | 0.44** | 0.38** | 0.39** | 0.34** |

**p < 0.01

**S4. Predictors of CDT factor scores: multiple regression analyses**

| **Predictor** | **CDT1 F1** | **CDT1 F2** | **CDT2 F1** | **CDT2 F2** |
| --- | --- | --- | --- | --- |
| MMSE orientation | 0.21** | 0.18* | 0.19* | 0.15* |
| MMSE memory | 0.24** | 0.20** | 0.22** | 0.19** |
| HDS-R verbal fluency | 0.23** | 0.19* | 0.21** | 0.18* |
| Adjusted R² | 0.32 | 0.27 | 0.29 | 0.21 |

*p < 0.05, **p < 0.01

**Note:** Standardized regression coefficients from exploratory forced-entry models.
